# Dietary Vitamin C Intake Is Associated With Improved Liver Function and Glucose Metabolism in Chinese Adults

**DOI:** 10.3389/fnut.2021.779912

**Published:** 2022-01-31

**Authors:** Xiaoqin Luo, Wanyu Zhang, Zhangya He, Hexiang Yang, Jiayi Gao, Pei Wu, Zheng Feei Ma

**Affiliations:** ^1^Department of Nutrition and Food Safety of School of Public Health, Xi'an Jiaotong University, Xi'an, China; ^2^Shaanxi Health Supervision Center, Xi'an, China; ^3^Emergency Medical Center, Xi'an Public Health Center, Xi'an, China; ^4^Department of Health and Environmental Sciences, Xi'an Jiaotong-Liverpool University, Suzhou, China

**Keywords:** vitamin C, CHNS, NAFLD, dietary, ascorbic acid

## Abstract

**Background:**

Non-alcoholic fatty liver disease (NAFLD) is one of the most prevalent chronic liver diseases worldwide. Dietary vitamin C intake might play an important role in reducing the risk of NAFLD. This study assesses the relationship between dietary vitamin C intake and diagnostic biomarkers of NAFLD.

**Methods:**

The data from the 2009 China Health and Nutrition Survey (CHNS), nine provinces across four diverse regions (Northeast, East Coast, Central, and West) were included in the study. The dietary vitamin C intake of participants was calculated based on 3-day 24-h diet questionnaires at the individual level. The associations of dietary vitamin C intake and the biochemical indicators of liver function and glucose/lipid metabolism were determined.

**Results:**

A total of 8,307 participants were included in the final analysis. The mean dietary vitamin C intake for the overall, male and female subjects was 79.8 ± 58.6, 81.6 ± 55.3, and 78.2 ± 61.2 mg/day, respectively. The prevalence of inadequate dietary vitamin C intake for the overall, male and female subjects was 24.4, 26.5, and 22.6%, respectively. Intake of vitamin C was associated with both lower concentrations of plasma ferritin and hemoglobin A1c (HbA1c). Higher dietary vitamin C intake was associated with higher albumin, even further adjusted for body mass index (BMI), residence, and smoking status. No improvement in lipid metabolism was found.

**Conclusion:**

This study demonstrated that higher dietary vitamin C intake is a benefit for improving glucose metabolism and liver function in which reducing ferritin, a biomarker of iron accumulation, may be involved.

## Introduction

Non-alcoholic fatty liver disease (NAFLD) is one of the most common causes of liver disease, which can progress to liver failure (1). NAFLD is caused by the accumulation of hepatic steatosis in individuals without excessive alcohol consumption (2, 3). The prevalence of NAFLD has increased significantly over the recent years, especially in individuals diagnosed with diabetes and obesity, both in the developed and developing regions. In China, NAFLD is one of the most prevalent chronic liver diseases, ranging from 6.2 to 38.2% (4).

Previous studies have reported that oxidative stress causes inflammation, which can impact chronic diseases, including NAFLD (5–9). Dietary factors including vitamin C may play an important role in reducing the risk of NAFLD. Vitamin C is an essential antioxidant that is capable of scavenging free radicals. In addition, it plays several important roles in enzymatic reactions as a reducing agent. Vitamin C has been suggested to be involved in regulating hepatic and circulating lipid homeostasis, providing evidence that vitamin C can be protective against nonalcoholic fatty liver disease (NAFLD). A study by Musso et al. reported that lower dietary vitamin C intake was reported in patients with nonalcoholic steatohepatitis (NASH) than healthy controls (10). Another study by Han et al. reported a positive relationship between low vitamin C intake and NAFLD in a Korean male population (11). Even though our recent randomized controlled trial of patients with NAFLD demonstrated an improvement in liver health and glucose metabolism after 12-week of vitamin C supplementation (12), the relationship between vitamin C and NAFLD remains controversial (13, 14).

In China, the average dietary vitamin C intake was lower than those reported in the European countries (15, 16). In addition, along with the rapid economic growth and urbanization, there have been dramatic changes in dietary patterns in the Chinese population (17). Dietary vitamin C intake has been reported to be associated with a reduced risk of NAFLD (18, 19). However, studies that have directly investigated the relationship of clinical indicators of patients with NAFLD and dietary vitamin C intake are few in number. Therefore, the aim of this study was to assess the relationship between dietary vitamin C intake and diagnostic biomarkers of liver function and glucose/lipid metabolism of NAFLD using the data from the China Health and Nutrition Survey (CHNS).

## Methods

### Study Population

**Figure 1:**
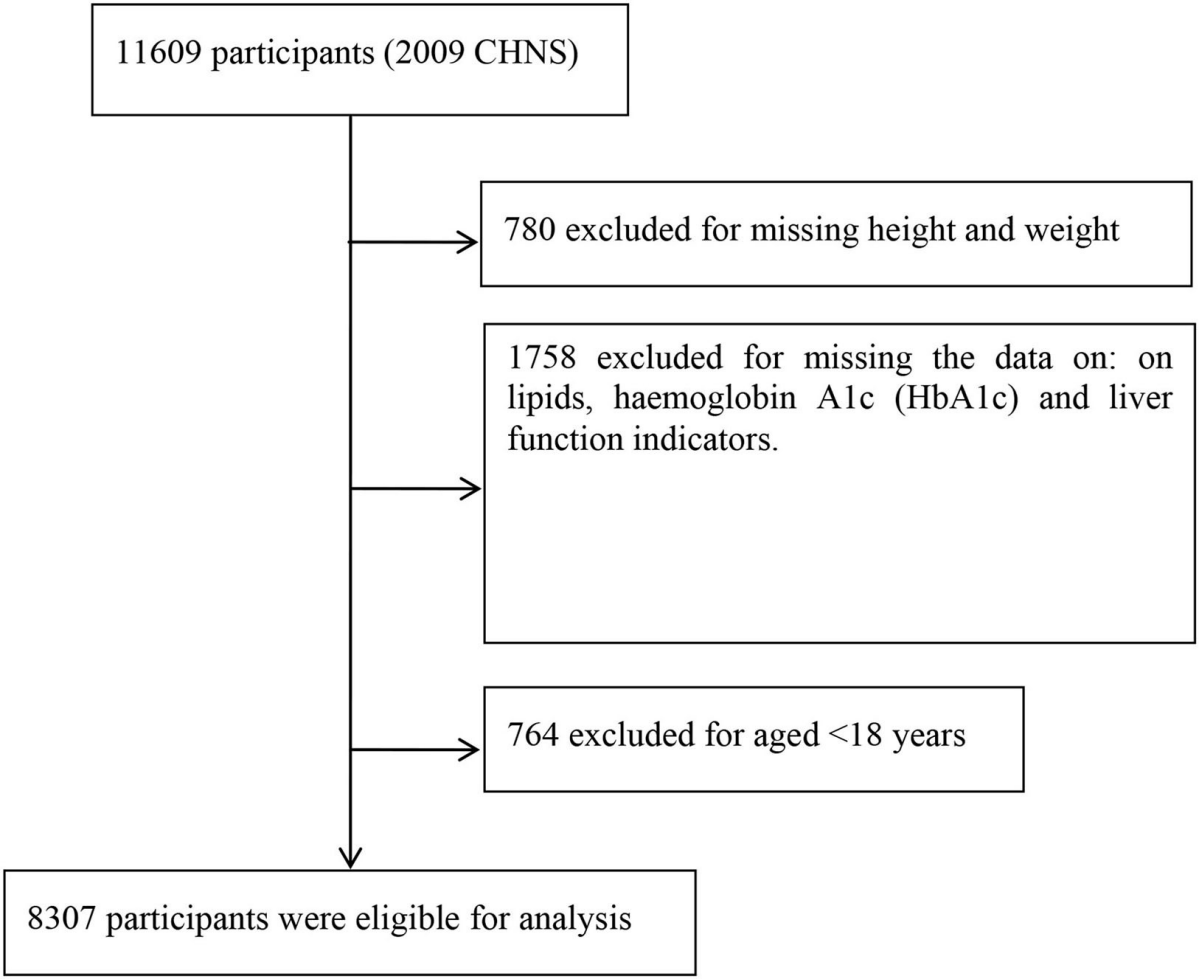
Flowchart of study participants.

We obtained the prospective longitudinal CHNS data that was designed to examine the effect of the health, nutrition, and family planning policies and programs implemented by the national and local governments, and to see how the social and economic transformation of Chinese society is affecting the health and nutritional status of its population. The details of the study design and sampling procedure have been described previously (20). The CHNS is an ongoing prospective study that started in 1989, with a follow-up every 2–4 years. A multistage, random cluster sampling process was used to draw a sample in each of the provinces in China. In each province, counties and cities were stratified by income, and a weighted sampling scheme was employed to choose four counties and two cities randomly from each province. We selected the 2009 survey population (*n* = 11,609) as the research population because their data of biomarkers of NAFLD by fasting blood were provided. Among them, 780 were excluded for missing height and weight information. A total of 1,758 were excluded for missing the data on lipids, hemoglobin A1c (HbA1c), and liver function indicators, and 764 were excluded for age <18 years (Figure 1). Finally, 8,307 participants were eligible for the analysis. This study had reviewed and approved by the Institutional Review Boards of the University of North Carolina, Chapel Hill, and the Chinese Center for Disease Control. All the participants provided written informed consent before participating in the CHNS survey.

### Nutrient Intake Calculation

According to the 3-day diet questionnaire, we obtained the type and amount of food consumed by each individual. We also established an electronic database of China Food consumption (2009) (21) by EpiData 3.1. The daily intake of various nutrients per person was calculated by the formula:


$$\begin{array}{l} \text{X}=\text{W}/100\times \text{EP}\times \text{A} \end{array}$$

(W: The average amount of food consumed per day; EP: Proportion of edible part of food in China Food consumption; A: The content of the nutrient of edible part of food in China food composition per 100 g).

The research population was divided into four groups according to the quartile of dietary vitamin C intake. The estimated dietary vitamin C intake was compared with the recommended nutrient intake (RNI) for vitamin C recommended by the Chinese Dietary Reference Intakes (DRIs), which is 100 mg/day (22). In addition, the proportion of participants with a vitamin C intake <100 mg/day was calculated and determined as the prevalence of inadequacy of vitamin C intake.

### Statistical Analysis

Continuous variables were expressed as mean ± SD and counting variables were expressed as number (percentage). Differences of characteristics among groups were assessed by one-way ANOVA for continuous variables and the chi-squared test for the categorical variables. General linear models were used to analyze associations of dietary vitamin C intake with biomarkers of liver function and glucose metabolism. Model 1 was adjusted for the dietary vitamin C intake, age, and gender. Model 2 was adjusted for model 1 variables in addition to BMI, residence, and smoking status. A *P* value < 0.05 was considered statistically significant. Analyses were performed using SPSS 25.0 for Windows (IBM SPSS, Chicago, IL, United States).

## Results

### General Characteristics of Participants

The general characteristics of 8,307 participants are given in Table 1. A total of 8,307 participants were divided into four groups by dietary vitamin C intake quartile. The ranges of dietary vitamin C intake in the lowest, the second, the third, and the highest groups were 0.00 to 46.11, 46.13 to 69.11, 69.13 to 99.16, and 99.16 to 991.30 mg/day, respectively. The mean dietary vitamin C intake for the overall, male and female subjects was 79.8 ± 58.6, 81.6 ± 55.3, and 78.2 ± 61.2 mg/day, respectively. The prevalence of inadequate dietary vitamin C intake for the overall, male and female subjects was 24.4, 26.5, and 22.6%, respectively. The percentage of male and urban residence increased across increasing intake quartile categories of dietary vitamin C. The average value of age and the distribution of BMI were significantly different in intake quartile groups of dietary vitamin C.

**Table 1:** General characteristics of the participants.

**Vitamin C** **intake**	**Q1** **(*n* = 2,076)**	**Q2** **(*n* = 2,077)**	**Q3** **(*n* = 2,077)**	**Q4** **(*n* = 2,077)**	***P* value**
**Median intake (mg/d)**	30.6 ± 11.3	57.8 ± 6.5	83.3 ± 8.7	147.4 ± 77.3	
**Age (years)**	52.22 ± 16.40	49.82 ± 15.08	50.18 ± 14.55	50.12 ± 14.07	<0.001
**Gender (n, %)**					
Male	891 (42.9%)	939 (45.2%)	1,002 (48.2%)	1,047 (50.4%)	<0.001
Female	1,185 (57.1%)	1,138 (54.8%)	1,075 (51.8%)	1,030 (49.6%)	
**Height (cm)**	160.32 ± 8.62	161.18 ± 8.60	161.34 ± 8.53	161.40 ± 8.49	<0.001
**Weight (kg)**	60.34 ± 11.51	60.91 ± 11.36	60.98 ± 11.22	61.11 ± 11.09	0.133
**BMI (n, %)**					
<18.5 kg/m^2^	147 (7.1%)	131 (6.3%)	138 (6.6%)	106 (5.1%)	0.341
18.5~23.9 kg/m^2^	1,101 (53.0%)	1,102 (53.1%)	1,102 (53.1%)	1,150 (55.4%)	
24.0~27.9 kg/m^2^	616 (29.7%)	634 (30.5%)	634 (30.5%)	628 (30.2%)	
≥28.0 kg/m^2^	212 (10.2%)	210 (10.1%)	203 (9.8%)	193 (9.3%)	
**Waistline (cm)**	83.25 ± 10.63	82.56 ± 10.44	82.54 ± 10.11	82.61 ± 10.26	0.083
**Hips (cm)**	94.44 ± 8.10	94.36 ± 7.99	94.32 ± 7.79	94.78 ± 7.65	0.212
**Residence (n, %)**					
Urban	597 (28.8%)	592 (28.5%)	711 (34.2%)	819 (39.4%)	<0.001
Rural	1,479 (71.2%)	1,485 (71.5%)	1,366 (65.8%)	1,258 (60.6%)	
**Smoking status (n, %)**					
Never	1,467 (70.7%)	1,458 (70.2%)	1,410 (67.9%)	1,398 (67.3%)	0.133
Former	67 (3.2%)	70 (3.4%)	62 (3.0%)	74 (3.6%)	
Current	541 (26.1%)	548 (26.4%)	604 (29.1%)	604 (29.1%)	

### Assessing the Association Between Dietary Vitamin C Intake and Biomarkers of Liver Function

**Figure 2:**
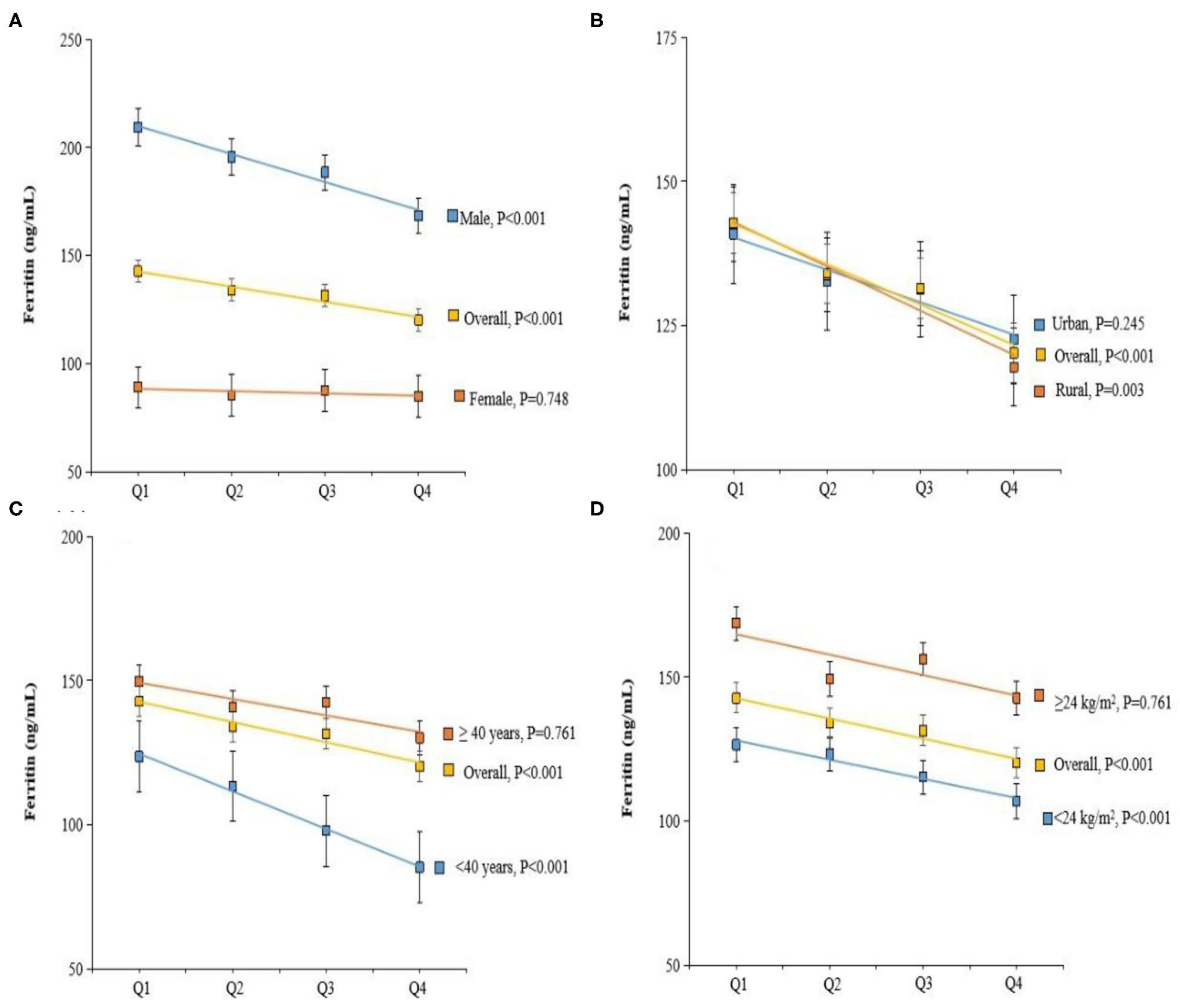
Adjusted Ferritin by dietary vitamin C intake, gender **(A)**, residence **(B)**, age **(C)**, and body mass index (BMI) **(D)**. Values were means adjusted for age, gender, BMI, smoking status, and residence.

The general linear model was constructed to assess whether dietary vitamin C intake was associated with plasma biomarkers of liver function. Intake of vitamin C was correlated with lower plasma ferritin concentrations (Table 2). The estimation means of plasma ferritin concentrations by the general linear model (adjustment for age, gender, and dietary vitamin C intake) was lower with the increase of the dietary vitamin C intake level, especially in the male, rural, age <40 y and BMI <24 kg/m^2^ participants (Figure 2). Higher dietary vitamin C intake was associated with higher albumin, even further adjusted for BMI, residence, and smoking status.

**Table 2:** Estimation mean (95% CI) of biomarkers of liver function according to dietary vitamin C intake (mg) among adults.

	**Q1** **(*n* = 2,076)**	**Q2** **(*n* = 2,077)**	**Q3** **(*n* = 2,077)**	**Q4** **(*n* = 2,077)**	**P-linear** **trend**
**Ferritin (ng/mL)**					
**Model 1**	151.6 (144.1, 159.1)	142.7 (135.2, 150.2)	140.5 (133.0, 148.0)	129.0 (121.5, 136.5)	0.001
**Model 2**	142.1 (132.0, 152.2)	133.2 (123.2, 143.3)	131.0 (121.0, 141.1)	119.5 (109.5, 129.5)	<0.001
**Transferrin (mg/dL)**					
**Model 1**	286.4 (284.1, 288.8)	289.3 (287.4, 292.1)	286.4 (284.0, 288.7)	287.7 (285.4, 290.0)	0.265
**Model 2**	286.3 (283.1, 292.3)	289.1 (286.0, 292.3)	286.4 (283.3, 290.0)	287.8 (284.6, 290.9)	0.293
**Soluble transferrin receptor (mg/L)**					
**Model 1**	1.45 (1.42, 1.48)	1.47 (1.44, 1.50)	1.46 (1.42, 1.49)	1.50 (1.46, 1.52)	0.148
**Model 2**	1.42 (1.38, 1.46)	1.44 (1.40, 1.48)	1.43 (1.39, 1.47)	1.48 (1.44, 1.52)	0.056
**Alanine transaminase (U/L)**					
**Model 1**	23.13 (22.34, 23.92)	23.14 (22.35, 23.93)	23.39 (22.60, 24.18)	22.69 (21.91, 23.49)	0.671
**Model 2**	22.03 (20.98, 23.08)	22.00 (22.95, 23.04)	22.38 (21.33, 23.42)	21.65 (20.62, 22.69)	0.639
**Total protein (g/L)**					
**Model 1**	74.79 (74.56, 75.01)	74.96 (74.74, 75.19)	74.92 (74.69, 75.14)	74.58 (74.36, 74.81)	0.089
**Model 2**	74.77 (74.46, 75.08)	74.94 (74.63, 75.24)	74.90 (74.60, 75.21)	74.56 (74.25, 74.86)	0.078
**Albumin (g/L)**					
**Model 1**	44.84 (44.69, 44.98)	45.06 (44.92, 45.20)	45.23 (45.09, 45.37)	45.25 (45.11, 45.39)	<0.001
**Model 2**	44.93 (44.74, 45.12)	45.15 (44.96, 45.34)	45.28 (45.09, 45.47)	45.26 (45.07, 45.45)	0.002

*Model 1 was adjusted for dietary vitamin C intake, age and gender; Model 2 was adjusted for Model 1 variables in addition to body mass index (BMI), residence, and smoking status*.

### Assessing Association Between Dietary Vitamin C Intake and Biomarkers of Glucose Metabolism

**Figure 3:**
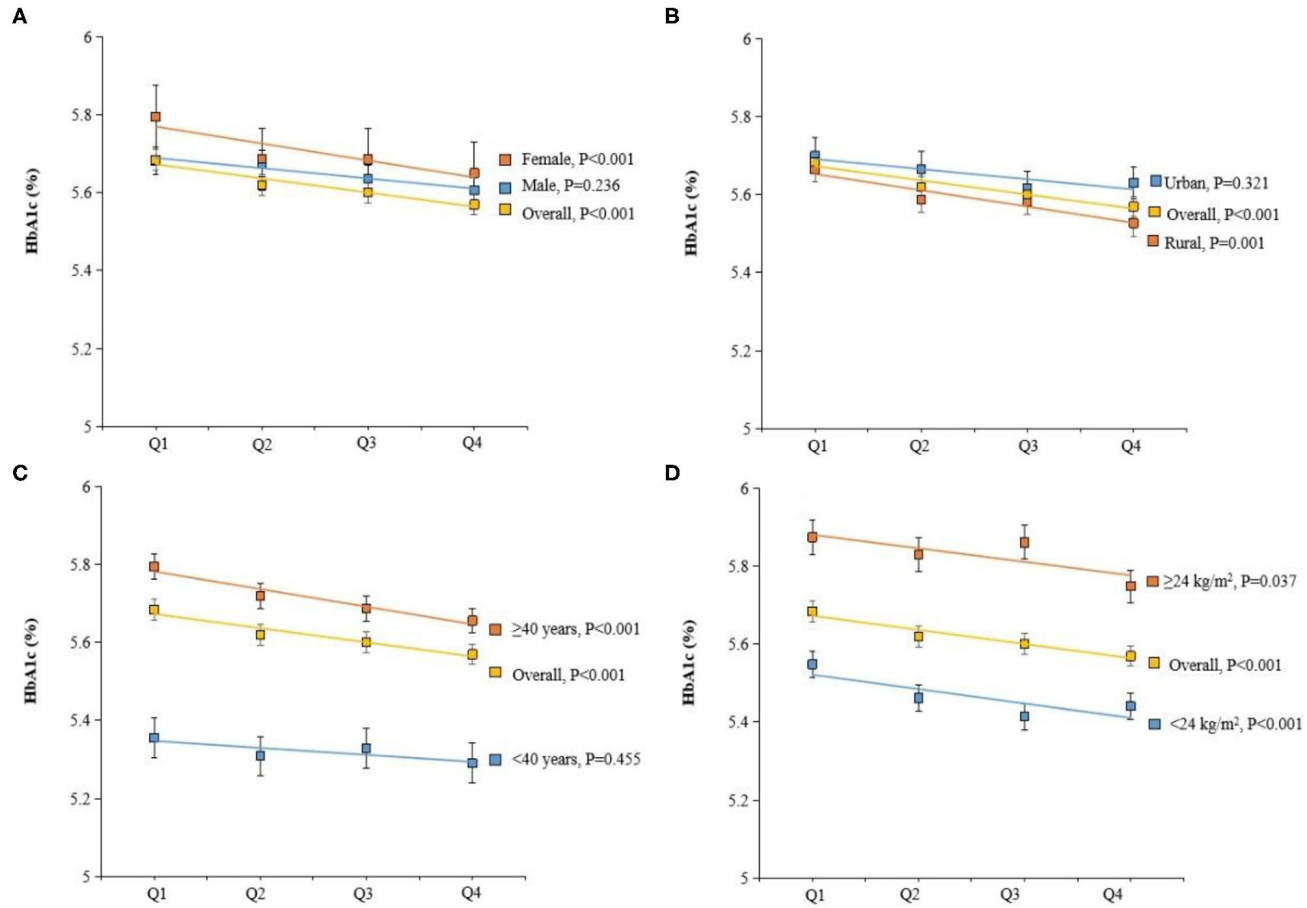
Adjusted hemoglobin A1c (HbA1c) (%) by dietary vitamin C intake, gender **(A)**, residence **(B)**, age **(C)**, and BMI **(D)**. Values were means adjusted for age, gender, BMI, smoking status, and residence.

Next, the general linear model was constructed to assess whether dietary vitamin C intake was associated with plasma biomarkers of glucose/lipid metabolism. Intake of vitamin C was correlated with lower plasma HbA1C concentrations (Table 3). The estimation means of plasma HbA1_C_ concentrations by the general linear model (adjustment for age, gender, and BMI) was lower with the increase of the dietary vitamin C intake level, especially in female, rural, and age <40 years participants (Figure 3). No significant changes in lipids, including total cholesterol, high-density lipoprotein cholesterol, low-density lipoprotein cholesterol, and total triglyceride were observed with the increase of dietary intake of vitamin C.

**Table 3:** Estimation mean (95% CI) of biomarkers of glucose/lipid metabolism according to dietary vitamin C intake (mg) among adults.

	**Q1** **(*n* = 2,076)**	**Q2** **(*n* = 2,077)**	**Q3** **(*n* = 2,077)**	**Q4** **(*n* = 2,077)**	**P-linear** **trend**
**Hemoglobin A1c (%)**					
**Model 1**	5.69 (5.65, 5.73)	5.63 (5.59, 5.67)	5.61 (5.58, 5.65)	5.58 (5.55, 5.62)	0.001
**Model 2**	5.69 (5.66, 5.73)	5.63 (5.59, 5.66)	5.61 (5.58, 5.65)	5.58 (5.54, 5.62)	0.001
**Blood glucose (mmol/L)**					
**Model 1**	5.40 (5.34, 5.46)	5.36 (5.29, 5.42)	5.42 (5.36, 5.48)	5.29 (5.23, 5.35)	0.014
**Model 2**	5.35 (5.26, 5.43)	5.30 (5.22, 5.38)	5.36 (5.28, 5.45)	5.22 (5.14, 5.30)	0.005
**Total cholesterol (mmol/L)**					
**Model 1**	4.89 (4.85, 4.93)	4.87 (4.82, 4.91)	4.88 (4.84, 4.92)	4.83 (4.78, 4.87)	0.186
**Model 2**	4.88 (4.82, 4.94)	4.86 (4.80, 4.92)	4.87 (4.82, 4.93)	4.82 (4.76, 4.87)	0.149
**Triglyceride (mmol/L)**					
**Model 1**	1.55 (1.49, 1.61)	1.63 (1.57, 1.69)	1.61 (1.55, 1.67)	1.58 (1.52, 1.63)	0.230
**Model 2**	1.53 (1.45, 1.61)	1.61 (1.53, 1.69)	1.60 (1.52, 1.67)	1.56 (1.48, 1.63)	0.187
**High-density lipoprotein cholesterol (mmol/L)**					
**Model 1**	1.45 (1.43, 1.47)	1.42 (1.40, 1.44)	1.42 (1.40, 1.44)	1.45 (1.43, 1.47)	0.071
**Model 2**	1.43 (1.40, 1.46)	1.40 (1.37, 1.43)	1.40 (1.38, 1.43)	1.43 (1.40, 1.46)	0.043
**Low-density lipoprotein cholesterol (mmol/L)**					
**Model 1**	3.01 (2.97, 3.05)	2.98 (2.94, 3.02)	2.98 (2.94, 3.02)	2.94 (2.90, 2.98)	0.206
**Model 2**	3.00 (2.94, 3.06)	2.98 (2.92, 3.03)	2.97 (2.92, 3.03)	2.94 (2.88, 2.99)	0.196

*Model 1 was adjusted for dietary vitamin C intake, age and gender; Model 2 was adjusted for model 1 variables in addition to BMI, residence, and smoking status*.

## Discussion

Vitamin C is a water-soluble vitamin, which is naturally present in fruits and vegetables. Since vitamin C cannot be synthesized by the human body, vitamin C is considered an essential dietary micronutrient and required to be obtained through diet. In China, the mean dietary vitamin C intake is estimated to be 80.1 mg/day (lower than the EAR of 85 mg/day). (17, 23). Similarly, our study also showed that the mean dietary vitamin C intake for the overall was almost 80 mg/day, suggesting that majority of the Chinese population have inadequate dietary vitamin C intake. Based on the evidence on its antioxidant properties, vitamin C is needed for reducing the risk of progression to some noncommunicable diseases including cardiovascular disease, type 2 diabetes, and liver diseases (24–26). A recent systematic review investigated that the efficacy of oral vitamin C supplementation significantly lowers HbA1c, fasting, and postprandial glucose in people with type 2 diabetes, highlighting that vitamin C supplementation may be beneficial for improving glycemic control in type 2 diabetes (27). However, these were not specific to people with NAFLD, and supplements were not exclusive to vitamin C only. Our recent randomized controlled study has also reported that oral vitamin C supplementation of 1,000 mg/day was associated with increased concentration of plasma vitamin C and adiponectin as well as improved glucose metabolism and liver function in patients with NAFLD. Furthermore, this study indicated a linear association between dietary vitamin C intake and liver function and glucose metabolism in the general population. Together, these studies suggest that vitamin C has a potentially important role in improving metabolism, warranting further evaluation to ascertain the potential clinical translatability of vitamin C supplementation from both diet and supplements.

This study examined the relationship between dietary vitamin C intake and biomarkers of liver function on a large sample comprising 8,307 adults. Several diagnostic biomarkers of early liver damage and NAFLD, such as total protein, albumin, and ALT, were included in our biochemical analysis. Patients with severe liver diseases usually have a lower concentration of albumin (28), which plays an important role in the clinical evaluation of liver function and is used as a diagnostic biomarker in the management of NAFLD (29). This is because, in patients with liver diseases including NAFLD, the synthesis of albumin by the liver is altered which leads to lower albumin concentration (28, 30). Our findings reported that higher dietary vitamin C intake was associated with higher albumin concentration, indicating that vitamin C is capable of increasing the reduced albumin of patients with NAFLD and then improving liver function.

Other indicators of liver function are those involved in iron metabolism. This study reported lower ferritin concentration with increasing dietary vitamin C intake. Iron is a trace element that reacts to oxygen radicals. Ferritin, a protein that contains iron, is one of the inflammatory biomarkers used in diagnosing NAFLD. A higher concentration of ferritin has been reported in steatohepatitis, which can be possibly attributed to systemic inflammation (31). A study by Sumuida et al. (32) reported increased ferritin concentration with increasing severity of NAFLD. This is because, in patients with NAFLD, the ferritin concentration has been reported to increase by 20–50%, which is suggested to be a useful biomarker for identifying individuals susceptible to NAFLD (33). This study suggested that vitamin C has a potential capacity of reducing circulating levels of ferritin, which may account for its antioxidant effect and improve NAFLD.

A remarkable proportion of patients with NAFLD also have a dysregulation of glucose metabolism (27). In this study, higher dietary vitamin C intake was associated with lower HbA1c concentration, consistently suggesting that supplementation of dietary vitamin C might decrease elevated HbA1c concentration in patients with NAFLD (11). In addition, our findings were also in agreement with the previously published data showing improvement in glycemic control with vitamin C supplementation (34). However, it is challenging to determine whether the beneficial effect of vitamin C supplementation could be attributed because of its correction of vitamin C deficiency status or effect on the underlying disease in individuals. A previous study reported that increased concentration of aminotransferases along with diabetes as independent predictors of fibrosis (35). Consistently, our finding that adequate dietary intake of vitamin C is beneficial in the improvement of glycemic control and the liver function in patients with NAFLD may give us a hint that the effect of vitamin C on fibrosis is also worthy of expectation.

One of the major strengths of this study was that it included a large sample size of the longitudinal data (CHNS). In addition, the use of validated individual, consecutive 3-day 24-h dietary recall method in this study had enabled us to thoroughly examine the detailed information of the foods and drinks consumed by the participants. Limitations of this study also merit consideration. Although we included the data presented on determining vitamin C content of food, we did not include the other co-occurring nutrients in the food discounted or adjusted for in the modeling. Since there were no data on the hepatitis status of participants included in the analysis, this might introduce bias in the interpretation of our study findings. Also, plasma vitamin C levels as a marker of the accuracy of the vitamin C intake determination were not measured in this study.

In conclusion, this study was one of the first studies to examine the relationship between dietary vitamin C intake and diagnostic biomarkers of NAFLD on a large sample with adjustment of confounding factors. Further understanding of this relationship would be helpful, especially in providing new clinical insights and implications on the roles of dietary vitamin C in the pathogenesis, management, and prevention of NAFLD.

## Data Availability Statement

Publicly available datasets were analyzed in this study. This data can be found here: https://www.cpc.unc.edu/projects/china.

## Ethics Statement

The studies involving human participants were reviewed and approved by Institutional Review Boards of the University of North Carolina, Chapel Hill, and the Chinese Center for Disease Control. The patients/participants provided their written informed consent to participate in this study.

## Author Contributions

XL and ZM reviewed and approved the study design and reviewed and revised the manuscript. All authors designed the statistical analysis plan, analyzed the data, reviewed and revised the results, agreed to be accountable for all the aspects of the study, read and approved the final version of the manuscript, conceptualized and designed the study, collected the data, and drafted the initial manuscript.

## Funding

This study was supported by the National Natural Science Foundation of China (No. 81874263). This study uses data from the China Health and Nutrition Survey (CHNS). We are grateful to research grant funding from the National Institute for Health (NIH), the Eunice Kennedy Shriver National Institute of Child Health and Human Development (No. R01 HD30880), National Institute on Aging (No. R01 AG065357), National Institute of Diabetes and Digestive and Kidney Diseases (Nos. R01DK104371 and R01HL108427), the NIH Fogarty grant (No. D43 TW009077) since 1989, and the China-Japan Friendship Hospital, Ministry of Health for support for CHNS 2009, Chinese National Human Genome Center at Shanghai since 2009, and Beijing Municipal Center for Disease Prevention and Control since 2011. The funding agency was not involved in the following tasks: research design and conduct; data collection, management, analysis, and interpretation; article preparation, review, or approval.

## Conflict of Interest

The authors declare that the research was conducted in the absence of any commercial or financial relationships that could be construed as a potential conflict of interest.

## Publisher's Note

All claims expressed in this article are solely those of the authors and do not necessarily represent those of their affiliated organizations, or those of the publisher, the editors and the reviewers. Any product that may be evaluated in this article, or claim that may be made by its manufacturer, is not guaranteed or endorsed by the publisher.
